# Demonstration of electron diffraction from membrane protein crystals grown in a lipidic mesophase after lamella preparation by focused ion beam milling at cryogenic temperatures

**DOI:** 10.1107/S1600576720013096

**Published:** 2020-10-13

**Authors:** Vitaly Polovinkin, Krishna Khakurel, Michal Babiak, Borislav Angelov, Bohdan Schneider, Jan Dohnalek, Jakob Andreasson, Janos Hajdu

**Affiliations:** a ELI Beamlines, Institute of Physics, Czech Academy of Science, Na Slovance 2, 18221 Prague, Czech Republic; b CEITEC – Central European Institute of Technology, Masaryk University, Kamenice 5/4, 62500 Brno, Czech Republic; c Institute of Biotechnology of the Czech Academy of Sciences, BIOCEV, Prumyslova 595, CZ-252 50 Vestec, Czech Republic; d Department of Cell and Molecular Biology, Uppsala University, Husargatan 3 (Box 596), SE-751 24 Uppsala, Sweden

**Keywords:** electron diffraction, membrane protein crystals grown in *meso*, lipidic cubic phases, focused ion beam milling, lamella preparation

## Abstract

Electron crystallography of sub-micrometre-sized 3D protein crystals has emerged recently as a valuable field of structural biology. *In meso* crystallization methods, utilizing lipidic mesophases, particularly lipidic cubic phases (LCPs), can produce high-quality 3D crystals of membrane proteins (MPs). A major step towards realizing 3D electron crystallography of MP crystals, grown *in meso*, is to demonstrate electron diffraction from such crystals. The first task is to remove the viscous and sticky lipidic matrix that surrounds the crystals without damaging the crystals. Additionally, the crystals have to be thin enough to let electrons traverse them without significant multiple scattering. In the present work, the concept that focused ion beam milling at cryogenic temperatures (cryo-FIB milling) can be used to remove excess host lipidic mesophase matrix is experimentally verified, and then the crystals are thinned to a thickness suitable for electron diffraction. In this study, bacteriorhodopsin (BR) crystals grown in a lipidic cubic mesophase of monoolein were used as a model system. LCP from a part of a hexagon-shaped plate-like BR crystal (∼10 µm in thickness and ∼70 µm in the longest dimension), which was flash-frozen in liquid nitro­gen, was milled away with a gallium FIB under cryogenic conditions, and a part of the crystal itself was thinned into a ∼210 nm-thick lamella with the ion beam. The frozen sample was then transferred into an electron cryo-microscope, and a nanovolume of ∼1400 × 1400 × 210 nm of the BR lamella was exposed to 200 kV electrons at a fluence of ∼0.06 e Å^−2^. The resulting electron diffraction peaks were detected beyond 2.7 Å resolution (with an average peak height to background ratio of >2) by a CMOS-based Ceta 16M camera. The results demonstrate that cryo-FIB milling produces high-quality lamellae from crystals grown in lipidic mesophases and pave the way for 3D electron crystallography on crystals grown or embedded in highly viscous media.

## Abstract 2

Electron diffraction experiments on crystals of membrane proteins grown in lipidic mesophases have not been possible owing to a thick layer of viscous crystallization medium around the crystals. Here it is shown that focused ion beam milling at cryogenic temperatures (cryo-FIB milling) can remove the viscous layer, and high-quality electron diffraction on a FIB-milled lamella of a bacteriorhodopsin 3D crystal is demonstrated.

## 1. Introduction   

Electron crystallography of 3D macromolecular crystals is a promising technique for solving structures at high resolution (Nannenga & Gonen, 2019 ▸). Profiting from strong interactions of electrons with matter (Henderson, 1995 ▸) and utilizing a standard cryo-electron microscope (cryo-EM) (Shi *et al.*, 2013 ▸, 2016 ▸), electron diffraction (ED) provides an effective means of solving structures of proteins and peptides from very thin crystals, which either would be difficult to study by synchrotron-based methods or can only be investigated with X-ray free-electron lasers (Neutze *et al.*, 2000 ▸; Chapman *et al.*, 2011 ▸; Wolff *et al.*, 2020 ▸). Moreover, atomic scattering factors for electrons, unlike those for X-rays, are strongly influenced by the charge state of the scattering atoms. This makes electron crystallography particularly suitable for mapping charged states of residues and cofactors of biomolecules and, in general, for investigating charge transfer and ion transport processes in biochemical reactions (Kimura *et al.*, 1997 ▸; Mitsuoka *et al.*, 1999 ▸; Subramaniam & Henderson, 2000 ▸; Gadsby, 2009 ▸; Yonekura *et al.*, 2015 ▸; Sjulstok *et al.*, 2015 ▸).

Since 2013, when the first protein structure was determined by the microcrystal electron diffraction technique (Shi *et al.*, 2013 ▸), 3D macromolecular crystallography with electrons has undergone significant developments in data collection and data analysis (Nannenga *et al.*, 2014 ▸; van Genderen *et al.*, 2016 ▸; Clabbers *et al.*, 2017 ▸; Gruene *et al.*, 2018 ▸; Hattne *et al.*, 2019 ▸; Bücker *et al.*, 2020 ▸), and a number of new protein and peptide structures have been solved (Rodriguez *et al.*, 2015 ▸; de la Cruz *et al.*, 2017 ▸; Xu *et al.*, 2019 ▸). For a typical ED data collection experiment, crystals in solution are pipetted onto a transmission electron microscopy (TEM) grid, blotted to remove excess solution, and vitrified in liquid ethane or nitro­gen. Finally, the diffraction data are recorded in a continuous rotation mode as the crystals are continuously rotated under parallel illumination with a high-energy electron beam (typically 200 kV) in a transmission cryo-EM (Hasegawa *et al.*, 2009 ▸; Shi *et al.*, 2016 ▸). The crystal thickness along the incident beam should be of sub-micrometre dimensions to reduce multiple scattering and absorption (Vulović *et al.*, 2013 ▸; Yan *et al.*, 2015 ▸; Clabbers & Abrahams, 2018 ▸; Latychevskaia & Abrahams, 2019 ▸).

Protein and peptide crystals below ∼0.5 µm thickness have successfully been used for structure determination (Rodriguez *et al.*, 2015 ▸; Sawaya *et al.*, 2016 ▸; Clabbers *et al.*, 2017 ▸; Xu *et al.*, 2019 ▸). Larger crystals can be broken up into smaller fragments by mechanical force (*e.g.* vigorous pipetting, sonication, vortexing with beads *etc*.) (de la Cruz *et al.*, 2017 ▸), but in this case one has little control over the final crystal size (Wolff *et al.*, 2020 ▸) and fragile crystals such as crystals of membrane proteins (MPs) (Michel, 1989 ▸; Moraes & Archer, 2015 ▸) may be damaged by mechanical processing. Alternatively, large protein crystals can be thinned in a more reproducible and reliable way to a desired size by focused ion beam milling under cryogenic conditions (cryo-FIB milling) (Duyvesteyn *et al.*, 2018 ▸; Zhou *et al.*, 2019 ▸; Martynowycz *et al.*, 2019*a* ▸). The latter technique has been inherited from materials science (Giannuzzi & Stevie, 1999 ▸) and cellular cryo-electron tomography (cryo-ET) (Marko *et al.*, 2007 ▸; Plitzko & Baumeister, 2019 ▸). Cryo-FIB milling enables ∼10 nm precision in the production of crystal lamellae of hundreds of nanometres in thickness and thus provides an important means of optimizing the ED signal (Zhou *et al.*, 2019 ▸). As cryo-FIB instrumentation becomes more accessible, this technique has the potential to become a standard tool, extending the capabilities of electron crystallography (Zuo & Spence, 1992 ▸; Stokes *et al.*, 2013 ▸; de la Cruz *et al.*, 2017 ▸; Nannenga & Gonen, 2019 ▸; Nannenga, 2020 ▸). We show here that cryo-FIB milling can overcome sample preparation challenges specific to membrane protein crystals grown in a viscous environment.

*In meso* crystallization of MPs utilizes lipidic mesophases (Ishchenko *et al.*, 2017 ▸; Caffrey & Cherezov, 2009 ▸), including lipidic cubic phases (LCPs) (Landau & Rosenbusch, 1996 ▸), where crystals grow in an extremely viscous medium, with viscosities of up to ∼50 Pa s (Perry *et al.*, 2009 ▸). The viscous crystallization medium cannot be easily removed from such crystals on TEM grids (Zhao *et al.*, 2019 ▸; Xu *et al.*, 2019 ▸), and this poses problems for electron diffraction due to the low penetration depth of electrons, which may be stopped in the bulky lipidic mesophase. The penetration depth of X-rays is orders of magnitude higher than that of electrons, and so the LCP technique has been successful in X-ray studies [see *e.g.* Pebay-Peyroula *et al.* (1997 ▸), Gordeliy *et al.* (2002 ▸), Cherezov *et al.* (2007 ▸) and Caffrey (2015 ▸)]. In contrast, not a single MP structure has been reported by electron diffraction from crystals grown *in meso*. The layer of the viscous crystallization medium can be several micrometres thick over the crystal, hindering the propagation of electrons. In principle, one can lower the viscosity of the LCP environment or dissolve it in a ‘chemical’ way by adding detergents (Luecke *et al.*, 1999 ▸), ‘spongifiers’ (Cherezov *et al.*, 2006 ▸) or oils (Niwa & Takeda, 2019 ▸) or by treating the LCP matrix with a lipase (Belrhali *et al.*, 1999 ▸) and then use standard blotting techniques to remove the liquid. This chemical approach was successfully demonstrated on non-membrane proteins, *e.g.* proteinase K crystals embedded in an LCP matrix (Zhu *et al.*, 2019 ▸). The chemical process is sample dependent and requires screening of phase-dissolving conditions to leave the particular type of crystal intact. For instance, the lipase treatment may degrade MP crystals by hydrolysis of lipid molecules, which interact with membrane proteins and stabilize the crystal structure (Hanson *et al.*, 2008 ▸; Liu *et al.*, 2012 ▸).

We demonstrate here that cryo-FIB milling at cryogenic temperatures has the potential to become a universal alternative to chemical cleaning. Conceptually, MP crystals grown in a lipidic mesophase are deposited (or grown directly) on a TEM grid, then vitrified by plunge-freezing, and the excess of lipidic crystallization matrix is milled away by the cryo-FIB. The thickness of the crystal can be adjusted to the desired level, to enable penetration of the electron beam and observation of the diffraction signal. This procedure can be general, unlike chemical cleaning, and could become a universal step in sample preparation for ED. The technique could potentially be applied to membrane or water-soluble protein crystals grown/embedded in a lipidic mesophase or, in general, in a viscous crystallization medium (*e.g.* bicelles, vesicles; Ishchenko *et al.*, 2017 ▸). Here, we report the first observed ED diffraction signal from a 3D MP crystal grown in a lipidic cubic phase. The crystal was cleaned up from the crystallization matrix and milled down to a thin lamella (∼210 nm, in this case) using the cryo-FIB.

## 2. Material and methods   

### 2.1. Protein crystallization   

Expression, purification and solubilization in β-octyl glucoside (Cube Biotech) and crystallization of bacteriorhodopsin (BR) (from *Halobacterium salinarum* S9 cells) in the lipidic cubic phase of monoolein (Nu-Chek Prep) were carried out as described by Gordeliy *et al.* (2003 ▸). Aliquots (200 nl) of a protein–mesophase mixture were spotted on a 96-well lipidic cubic phase glass sandwich plate (Marienfeld) and overlaid with 800 nl of precipitant solution by means of an NT8 crystallization robot (Formulatrix). Na/K phosphate (pH 5.6) solutions at concentrations from 1 to 2.4 *M* (in concentration of phosphate) were used as precipitant. Hexagonally shaped plate-like BR crystals of space group *P*6_3_ were observed after 2–3 weeks at room temperature.

### 2.2. Sample preparation for ED experiments by cryo-FIB milling   

**Figure 1:**
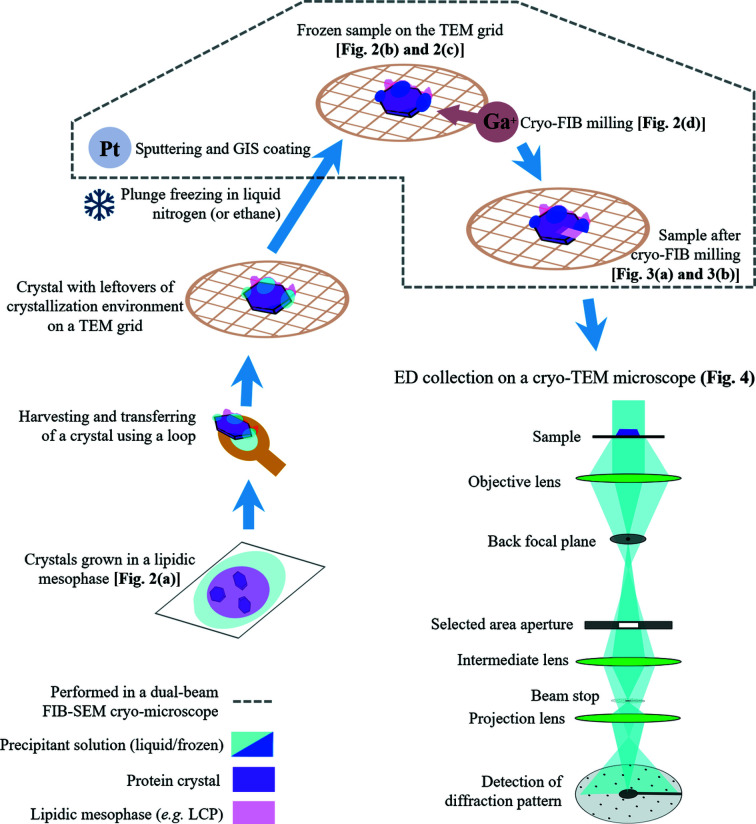
Schematic diagram of the experimental workflow. Schematic view of the electron diffraction setup is adapted from Rodriguez & Gonen (2016 ▸).

**Figure 2:**
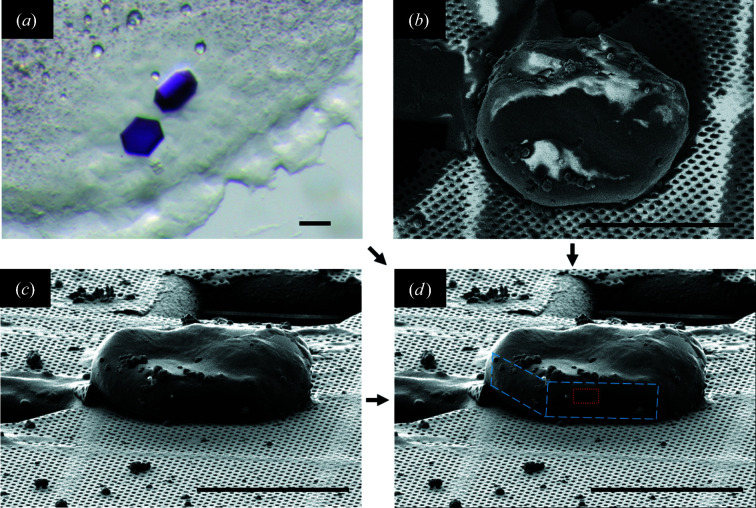
Images of bacteriorhodopsin crystals before cryo-FIB milling. Bright-field optical micrograph (*a*) of BR crystals grown in an LCP of monoolein at room temperature. SEM (*b*) and FIB (*c*) micrographs of a flash-frozen BR crystal with leftovers of the crystallization medium. (*d*) Geometrical features of the hexagon-shaped BR crystal are indicated by blue dashed line on the FIB image. These features can be guessed by comparing (*a*), (*b*) and (*c*). The red-dashed-line rectangle in (*d*) shows the selected area for further cryo-FIB milling. The scale bars correspond to 50 µm.

**Figure 3:**
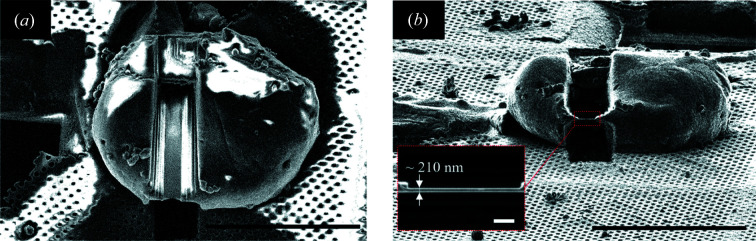
Images of the bacteriorhodopsin crystals after cryo-FIB milling. SEM image (*a*) and FIB image (*b*) of the final lamella obtained by the cryo-FIB milling. The inset shows the zoomed image of the lamella. The scale bars correspond to 50 µm for (*a*) and (*b*) and to 1 µm for the inset in (*b*).

The sample preparation workflow is shown schematically in Fig. 1 ▸. The crystals were harvested directly from the LCP matrix [Fig. 2 ▸(*a*)] using a MiTeGen microloop, then positioned on a holey carbon TEM grid (Quantifoil R2/1, Cu, 200-mesh) pre-clipped into an Autogrid assembly (Thermo Fisher Scientific) and manually plunge-frozen in liquid nitro­gen. The frozen sample was transferred into a dual-beam focused ion beam scanning electron microscope (Versa 3D, Thermo Fisher Scientific), equipped with a Quorum PP3010T cryogenic sample preparation–transfer system. The specimen was kept at 81 K on the cryo-stage during the procedure. The lamella preparation protocol followed approaches published earlier (*e.g.* Schaffer *et al.*, 2017 ▸; Duyvesteyn *et al.*, 2018 ▸; Martynowycz *et al.*, 2019*b* ▸; Zhou *et al.*, 2019 ▸). The crystal was coated with a conductive Pt layer by sputtering (10 mA, 60 s) and a protective organometallic layer of methylcyclopentadienyl (trimethyl) platinum with the help of a gas injection system (303 K, 80 s). Scanning electron microscopy (SEM) [Fig. 2 ▸(*b*)] and FIB imaging [Fig. 2 ▸(*c*)] were used to monitor the milling process and were performed with a 5 kV, 27 pA electron beam and a 30 kV, 10 pA gallium ion (Ga^+^) beam. The crystal was milled by rastering over the area of interest [Fig. 2 ▸(*d*)] by a 30 kV Ga^+^ beam, slowly removing portions of the sample above and below the selected area. Milling was performed in a stepwise way, reducing the beam current as the crystal lamella was thinned down to a final thickness of ∼210 nm (Fig. 3 ▸). Currents of 1 nA, 300 pA, 100 pA, 30 pA and 10 pA were used (in this order) to generate a final lamella by decreasing the thickness of the sample to ∼5 µm, ∼2 µm, ∼1 µm, ∼400 nm and all the way to the final thickness of ∼210 nm, respectively. The lamella was produced at an angle of 22° with respect to the grid plane. This orientation was selected for FIB milling because some of the edges of the hexagon-shaped BR crystal became visible at this angle [Fig. 2 ▸(*d*)], and this helped us to follow the process of FIB milling. The grid with the BR crystal lamella was subsequently transferred to a TEM microscope (Talos Arctica, Thermo Fisher Scientific) for the collection of the ED data.

### 2.3. Electron diffraction data collection and analysis   

**Figure 4:**
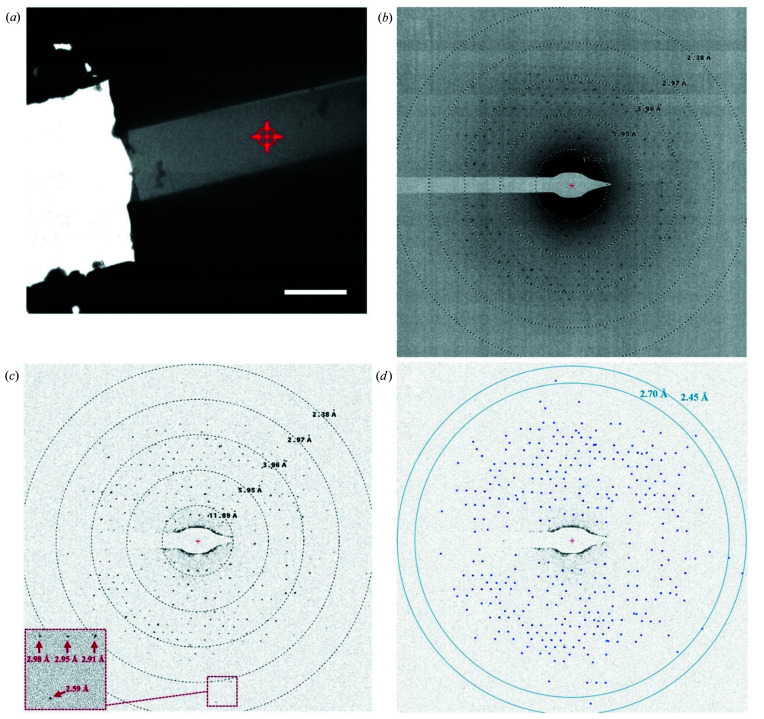
Electron diffraction experiment on a 210 nm-thick lamella of bacteriorhodopsin, using a 200 kV cryo-TEM microscope. (*a*) TEM micrograph of the FIB-machined lamella of the BR crystal. The electron diffraction signal was collected from a 1.4 µm area of the lamella, indicated by a red circle with a cross. The scale bar in (*a*) corresponds to 5 µm. (*b*) The 200 kV electron diffraction pattern obtained from the area indicated in (*a*). (*c*) The electron diffraction image is corrected by subtraction of a local moving-average background, calculated with the *Adxv* program (https://www.scripps.edu/tainer/arvai/adxv.html). The inset shows a close-up of the electron diffraction pattern. (*d*) Diffraction peaks were automatically picked up by the *Adxv* software, and the diffraction peaks in the resolution shell of 2.7–2.45 Å had an average peak height to background ratio of 2.5.

We used a Talos Arctica cryo-EM (Thermo Fisher Scientific), equipped with a Schottky field emission gun (XFEG) and operated at 200 kV, corresponding to an electron wavelength of 0.02508 Å. The crystal lamella was identified in imaging mode [Fig. 4 ▸(*a*)] at a low magnification and at a low dose rate. The ED experiment was performed under parallel illumination conditions utilizing microprobe mode and spot size 11, resulting in an illuminated area of ∼4.6 µm in diameter on the sample plane. Under these conditions the flux for the electron beam was ∼0.006 e Å^−2^ s^−1^ (derived from a recorded fluence of ∼0.06 e Å^−2^ during a 10 s exposure). ED data were collected from a crystal lamella zone of 1.4 µm in diameter as defined by a selected area aperture. ED data were recorded as a still image with a CMOS-based Ceta 16M detector (Thermo Fisher Scientific), using 2 × 2 binning and a 10 s exposure. The effective sample-to-detector distance was 2767 mm, as calibrated using a standard gold-film sample. For the evaluation of the ED pattern shown in Fig. 4 ▸(*b*), we calculated the ratio of the maximum peak intensity and the average local background in a square box of 20 by 20 pixels, excluding the peak. This was done for all peaks in the outermost resolution shell of 2.7–2.45 Å [Fig. 4 ▸(*d*)]. The ratios were averaged over all the peaks within the shell, giving the ‘average peak height to background ratio’ for the shell.

## 3. Results and discussion   

### 3.1. Crystal lamella preparation   

BR (Oesterhelt & Stoeckenius, 1971 ▸; Lanyi, 2004 ▸) was used as a model system because it is one of the most well characterized MPs and has been instrumental for the development of a vast array of biophysical techniques, including *in meso* crystallization methods (Landau & Rosenbusch, 1996 ▸; Faham & Bowie, 2002 ▸; Takeda *et al.*, 1998 ▸; Pebay-Peyroula *et al.*, 1997 ▸), and used in cryo-electron microscopy and 2D electron crystallography of proteins (Henderson *et al.*, 1990 ▸; Kimura *et al.*, 1997 ▸; Mitsuoka *et al.*, 1999 ▸; Subramaniam & Henderson, 2000 ▸). BR crystals were grown in an LCP of monoolein to a size of ∼60–80 µm in the longest dimension and to a thickness of ∼5–10 µm [Fig. 2 ▸(*a*)] by a standard LCP method (Pebay-Peyroula *et al.*, 1997 ▸; Gordeliy *et al.*, 2003 ▸; Polovinkin *et al.*, 2014 ▸), which reproducibly produces the hexagon-shaped plate-like crystals of type I and space group *P*6_3_. One of the crystals was used for lamella preparation as shown schematically in Fig. 1 ▸.

Fig. 2 ▸ shows images of the crystals before milling. We were able to identify some of the edges of the hexagon-shaped BR crystal in the FIB image [Fig. 2 ▸(*d*)] despite the presence of leftovers of the frozen LCP matrix and the precipitant solution.

We expect that, in general, it would be a difficult task to find an appropriate area on the crystal to mill if the navigation were based on FIB (or SEM) images, containing only the topographical information about the sample. Furthermore, locating crystals can be even more challenging if all the geometrical features of the crystals are hidden in a bulky crystallization matrix. The problem is likely to become even more pronounced if the crystals are only a few micrometres in size or smaller, and are buried in tens of micrometres of crystallization matrix [*e.g.* see figures published by Liu *et al.* (2013 ▸, 2014 ▸) and Zhu *et al.* (2019 ▸)]. In principle, crystals can be localized in a bulky sample by correlative 3D light microscopies in a nondestructive way and with micrometre or sub-micrometre precision, prior to or in parallel to FIB milling. The localization can be based on single- or multi-photon fluorescence (Judge *et al.*, 2005 ▸; Svoboda & Yasuda, 2006 ▸; Madden *et al.*, 2011 ▸; Leung & Chou, 2011 ▸; Jonkman & Brown, 2015 ▸; Li *et al.*, 2015 ▸), second harmonic generation (Kissick *et al.*, 2010 ▸; Wampler *et al.*, 2008 ▸) or the Raman effect (Aksenov *et al.*, 2002 ▸; Pezacki *et al.*, 2011 ▸; Hazekamp *et al.*, 2011 ▸; Opilik *et al.*, 2013 ▸; Arzumanyan *et al.*, 2016 ▸). Moreover, correlative cryo-fluorescence 3D light microscopy is currently a part of the cellular cryo-ET workflow (Plitzko & Baumeister, 2019 ▸; Schaffer *et al.*, 2019 ▸). This type of instrumentation and the methods outlined create an efficient basis for site-specific cryo-FIB milling of bulky samples containing target protein crystals.

Following the removal of excess crystallization matrix, the selected part of the BR crystal [Fig. 3 ▸(*a*)] was thinned down to ∼210 nm thickness [Fig. 3 ▸(*c*)] using focused 30 kV Ga^+^ ions. The obtained thickness is within the optimal range recommended for ED signal optimization (∼150–250 nm) in other 200 kV electron crystallography experiments with FIB-machined lysozyme crystal lamellae of various thicknesses (Zhou *et al.*, 2019 ▸). However, considering the ED signal, 210 nm might not be the optimal thickness for BR crystals. In general, the optimal crystal thickness for ED data collection depends on multiple factors. These factors include (i) the electron energy, which determines the elastic and inelastic mean-free paths and multiple scattering effects in a given sample (Clabbers & Abrahams, 2018 ▸; Latychevskaia & Abrahams, 2019 ▸); (ii) the effective sample thickness, which is a function of tilt angle during data collection (Plitzko & Baumeister, 2019 ▸); (iii) the mechanical stability of the sample which influences handling and transportation (Plitzko & Baumeister, 2019 ▸; Zhou *et al.*, 2019 ▸); (iv) the balance between the kinematic Bragg intensities (these are nearly proportional to the thickness squared) and the thickness-dependent corruptions of intensities, introduced by absorption and multiple scattering effects (Clabbers & Abrahams, 2018 ▸; Latychevskaia & Abrahams, 2019 ▸); and (v) the bulk-solvent content of the protein crystal, which modulates the gross effect of multiple scattering (Michel, 1989 ▸; Weichenberger *et al.*, 2015 ▸; Latychevskaia & Abrahams, 2019 ▸). Therefore, the thickness optimal for ED data collection can differ in each particular case. The easiest way of finding the optimal thickness could be to collect and to compare continuous rotation ED data at different thicknesses for different electron energies, which is a goal set for future studies.

### 3.2. The electron diffraction experiment   

Upon irradiating the 210 nm machined lamella [Fig. 3 ▸(*b*) and 3 ▸(*c*)] by a parallel 200 kV electron beam at a fluence of 0.06 e Å^−2^ and accumulating the ED signal from the 1.4 µm-diameter [Fig. 4 ▸(*a*)] zone, we observed an ED signal with an average peak height to background ratio of 2.5 for peaks in the 2.7–2.45 Å resolution shell (Fig. 4 ▸). Assuming an overall fluence of ∼2 e Å^−2^ (for 200 kV electrons) as ‘damage safe’ (as observed in experiments with 200–400 nm-thick crystals of proteinase K; Hattne *et al.*, 2018 ▸), we could roughly estimate that a wedge of around 15–30° can be collected from a single BR crystal lamella, when continuously rotating it with a fluence of 0.06 e Å^−2^ per frame and 0.5–1° rotation per frame [often used for ED data collection with scintillator-coupled electron detectors (Nannenga, 2020 ▸), such as the CMOS-based Ceta 16M camera used in the present study]. Taking into account the fact that BR crystals belong to the space group *P*6_3_, one would expect that a ∼90° wedge needs to be collected to get a data set resulting in a good completeness (Dauter, 1999 ▸; Borshchevskiy *et al.*, 2011 ▸). This implies that, in order to collect ED data suitable for high-resolution structural determination with the experimental system used in the present article, at least 3–6 different lamellae have to be produced and have to survive transfer from the cryo-FIB instrument to the cryo-TEM microscope. Cryo-FIB milling is time consuming (∼5 h per lamella in our case) and the transfer-survival rate was about ∼50%. We believe that the success rate could be improved significantly with practice [see *e.g.* Medeiros *et al.* (2018 ▸)].

We note that the experimental system that was available to us was not optimized for diffraction experiments. In particular, the CMOS-based detector Ceta 16M had a quantum efficiency of only 0.09 at 1/2 Nyquist frequency. Direct electron detectors have much higher sensitivity (McMullan *et al.*, 2016 ▸; Naydenova *et al.*, 2019 ▸; van Genderen *et al.*, 2016 ▸) and would allow significant improvements in data quality and resolution, enabling experimenters to collect data over a much wider angular range and at much improved signal-to-noise ratios (Hattne *et al.*, 2019 ▸). Such detectors would permit higher-resolution ED data while also minimizing the number of lamellae necessary to reach good completeness. Note also that with proficient users the success rate of transferring cryo-FIB lamellae of cells from a cryo-FIB/SEM microscope to a cryo-TEM microscope has been reported to reach 90% (Medeiros *et al.*, 2018 ▸). With more experience in sample handling and the availability of a direct electron detector suitable for ED experiments, we believe that only a few cryo-FIB crystal lamellae would be needed for solving the structure of BR or similar membrane proteins.

In another experiment, using a BR crystal lamella similar to the one reported here but of lower quality (diffraction to about 4 Å), we collected ED diffraction data over an angular range of 30° in a continuous rotation mode. The unit-cell dimensions were *a* = *b* = 60.64, *c* = 109.86 Å, α = β = 90, γ = 120°, in the space group *P*6_3_ (see Table S1), when the data were indexed and integrated by the software *XDS* (Kabsch, 2010 ▸) and then treated by the software *AIMLESS* (Evans & Murshudov, 2013 ▸) for scaling and merging. This result is consistent with values observed in X-ray diffraction experiments (Luecke *et al.*, 1999 ▸; Borshchevskiy *et al.*, 2011 ▸; Polovinkin *et al.*, 2014 ▸).

### 3.3. Practical aspects of lamella preparation   

We found that it was important to use a low FIB current of 10 pA at the final step of milling when removing the last ∼100 nm from each side of the lamella (see Section 2.2). In a few experiments, when we tried to minimize the FIB-milling time, we used a 30 pA current instead of 10 pA in the final step. Although the milling time decreased from ∼60 to ∼20 min, the use of the higher current led to lower-quality lamellae, diffracting to 7–4 Å only. Of course, this could have been caused by other factors such as crystals grown to lower quality or damaged during transfer from the crystallization probe to a TEM grid. Nevertheless, similar observations were reported by Martynowycz *et al.* (2019*b* ▸) in experiments on FIB-milled lamellae of proteinase K crystals. The authors utilized a cryo-FIB instrument similar to that used in our present study and showed that focused Ga^+^ beams with 10 and 30 pA current produced lamellae of different qualities, diffracting to 1.8 and 2.1 Å, respectively. We can speculate that local heating induced by higher currents can provoke disordering or devitrification processes, which are less pronounced in the case of lower currents [see Marko *et al.* (2006 ▸) and Plitzko & Baumeister (2019 ▸)]. These and similar observations suggest that milling conditions at cryogenic temperatures should be mapped out for each type of crystal to find a suitable balance between diffraction quality and overall milling time. Crystals of macromolecules can have significantly different water content and crystallization medium (Michel, 1989 ▸; Weichenberger *et al.*, 2015 ▸), and this may influence the results. FIB milling can be significantly accelerated by using ions other than Ga^+^. For example, new plasma FIBs utilizing noble gas ions promise removal rates that are up to 60× faster than the rate achievable with Ga^+^ ions (Marko *et al.*, 2006 ▸). The potential of utilizing FIBs with different ions for macromolecular electron crystallography has yet to be explored.

## 4. Conclusion   

Sample preparation is a critical and onerous step in both electron microscopy and crystallography of macromolecules. We have presented a sample preparation scheme, using FIB milling under cryogenic conditions, for electron crystallography for studies on 3D crystals of membrane proteins grown *in meso*. Our results demonstrate that a lamella of high quality for ED studies can be prepared from a 3D MP crystal surrounded by a highly viscous and sticky lipidic cubic mesophase. The cryo-FIB milling technique gives precise control over the thickness of crystal lamellae, which potentially permits ED data optimization in terms of resolution and influence of multiple scattering. This can lead to more accurate kinematic structure factors and the precise location of ions and hydrogen atoms in the electrostatic potential maps (Yonekura *et al.*, 2015 ▸; Palatinus *et al.*, 2017 ▸; Clabbers *et al.*, 2019 ▸), which can be crucial for understanding vital processes run by MPs.

We believe that the cryo-FIB milling method could be applicable to crystals of membrane or non-membrane proteins grown or embedded in various lipidic mesophases, including bicells and vesicles, or in other viscous crystallization media such as concentrated polyethyl­ene glycol solutions and media used for high-viscosity injectors [*e.g.* media used by Weierstall *et al.* (2014 ▸) and Wolff *et al.* (2020 ▸)]. Moreover, we believe that the cryo-FIB milling technique could also be suitable for crystals of few micrometres or even of smaller sizes. 3D electron micro-/nanocrystallography of MP crystals grown *in meso* could be facilitated by correlative 3D light microscopies, which could help to localize very small crystals in a bulky crystallization environment for subsequent cryo-FIB milling.

## Supplementary Material

Raw image of the electron diffraction pattern depicted in Fig. 4(b). DOI: 10.1107/S1600576720013096/te5063sup1.tif

Supporting information file. DOI: 10.1107/S1600576720013096/te5063sup2.pdf
