# Supplementary material for: Demonstration of electron diffraction from membrane protein crystals grown in a lipidic mesophase after lamella preparation by focused ion beam milling at cryogenic temperatures
Source: J Appl Crystallogr. 2020 Oct 13;53(Pt 6):1416–24. doi: 10.1107/S1600576720013096 (PMC7710488; doi:10.1107/S1600576720013096)
Supplement: Supplementary file 2 [file j-53-01416-sup2.pdf]

Table S1 Electron diffraction data were recorded in a continuous rotation mode from a single cryo-FIB lamella of a BR crystal. The data were indexed and integrated by the software XDS (Kabsch, 2010) and then treated by the software AIMLESS (Evans & Murshudov, 2013) for scaling and merging.

Data Collection	
Accelerating voltage	200 kV
Electron Source	field emission gun
Wavelength (Å)	0.0251
Frame rate (s^{-1})	1
Total number of frames	60
Rotation speed (°/s)	0.5
Fluence ($e^-/Å^2/s$)	0.06
Effective detector distance (mm)	2215
Data Processing	
Space group	$P6_3$
Unit Cell	
a, b, c (Å)	60.64, 60.64, 109.86
α, β, γ (°)	90, 90, 120
Resolution (Å)	19.9-4.2<br>(4.7-4.2)
Total reflections	3130 (937)
Multiplicity	4.5 (4.7)
Completeness (%)	39.1 (40.4)
$CC_{1/2}$	0.916 (0.408)
R_{meas}	0.448 (0.623)
R_{merge}	0.359 (0.5)
$\langle I/\sigma I \rangle$	3.0 (2.5)
